# Perampanel’s forgiveness factor in a variable medication adherence paradigm in a rat model of chronic epilepsy

**DOI:** 10.1186/s12967-023-04490-z

**Published:** 2023-09-20

**Authors:** Michelle Guignet, Amanda Campbell, Jonathan Vuong, Dale Whittington, H. Steve White

**Affiliations:** 1 School of Pharmacy Seattle, University of Washington, Seattle, WA USA; 2 Center for Epilepsy Drug Discovery, Department of Pharmacy, School of Pharmacy, University of Washington, Health Sciences Building F563, 1959 NE Pacific Street, Box 357630, Seattle, WA 98195-7630 USA; 3 Department of Medicinal Chemistry, School of Pharmacy, University of Washington, Seattle, WA 98159 USA

**Keywords:** Adherence, Epilepsy, Antiseizure medicine, Perampanel, Breakthrough seizures

## Abstract

**Background:**

Poor medication adherence contributes to increased morbidity and mortality in patients with epilepsy and may be under-addressed in clinical practice. Ethical concerns make it impossible to study the impact of medication nonadherence in clinical trials, but our previous work emphasizes the importance of using preclinical approaches to address these questions. With over 30 clinically available antiseizure medicines (ASM’s), it remains an important question to understand the relationship between poor adherence and seizure incidence across mechanistically distinct ASM’s, including the broad-spectrum ASM, perampanel (PER).

**Methods:**

We formulated PER into chow pellets to deliver to rats in a 100% fully adherent or 50% variable nonadherent paradigm via our novel automated medication-in-food delivery system. Chronic oral dosing was initiated in male rats with chronic epilepsy while monitoring 24/7 for videoEEG evidence of seizures during a 4-week placebo baseline and 4-week treatment phase. PER concentrations were monitored in plasma at 1-week intervals and correlated with degree of seizure control. The relationship between missed doses and extended patterns of nonadherence were correlated with breakthrough seizures.

**Results:**

Fully adherent rats demonstrated a median reduction in seizure frequency of 50%, whereas nonadherent rats had a median increase of 54%. Plasma concentrations of PER were stable over the 4-week treatment period in both fully adherent and nonadherent groups, with levels being twice as high in fully adherent animals. There was no correlation between a single missed dose or series of missed doses and the incidence of breakthrough seizures. However, those animals in the nonadherent group that received PER for every meal during a 24-h period had a reduced likelihood of seizure incidence.

**Conclusions:**

If our preclinical data is supported in the clinic, PER’s favorable pharmacokinetic profile in humans, combined with a lowered risk of breakthrough seizures suggests that it may provide a certain forgiveness factor if a dose is missed within a 24-h window.

**Supplementary Information:**

The online version contains supplementary material available at 10.1186/s12967-023-04490-z.

## Introduction

One third of people living with epilepsy cannot obtain adequate seizure control with their antiseizure medicine (ASM) [1]. However, uncontrolled seizures do not necessarily equate to drug-resistant epilepsy. It is possible that poor adherence, or the extent to which a person adheres to their prescribed regimen, may negatively impact seizure control and lead to increased morbidity and mortality [2]. Estimates vary but it is thought that up to 50–75% of adults with epilepsy demonstrate some degree of suboptimal adherence (e.g., < 80%) (see [3] for review). Reasons for not taking medication can be complex and vary from person to person, demonstrating that adherence is a dynamic health behavior that is significantly influenced by socioeconomic, demographic, and therapy-related (e.g., side effects, complexity of prescribing regimens) factors [4]. Regardless, the clinical implications of adjusting pharmacotherapy in response to breakthrough seizures (e.g., therapy change or dose escalation) without first addressing the underlying issues with adherence are not fully understood.

Given that clinical studies are time-consuming, costly, and unethical to perform for these types of questions, the availability of animal models has furthered our understanding of the direct relationship between nonadherence and seizure burden. Previous work in our lab utilized a clinically relevant, novel medication-in-food delivery system in a rat model of temporal lobe epilepsy (TLE) to demonstrate that correcting for nonadherence to the ASM, carbamazepine (CBZ), improves seizure control better than changing pharmacotherapy (i.e., dose escalation) [5–7]. Moreover, these studies highlight that a single missed dose of CBZ can increase the risk of seizure days after the missed dose occurred (even if subsequent doses have been taken), suggesting that factors beyond a drug’s pharmacokinetics may impact seizure control. Notably, these studies were only completed using a single ASM (e.g., CBZ). With over 30 clinically available ASM’s with distinct pharmacokinetic properties and mechanisms of action [8], it remains an important question to expand upon these results with CBZ to better understand how mechanistically distinct ASM’s impact seizure control in a variable medication adherence paradigm.

Perampanel (PER) is a mechanistically novel ASM that works to prevent seizure initiation and spread at the level of postsynaptic AMPA glutamate receptors [9]. Since its approval in 2012 for adjunctive treatment of focal-onset seizures [10], real world and open-label studies have since supported PER’s favorable efficacy and tolerability profile as monotherapy/first-line adjunctive therapy for focal onset and generalized tonic–clonic seizures [11]. Moreover, the fact that PER is a once daily oral tablet with a long half-life (t_1/2_ = 105 h, [12]) may allow for prolonged therapeutic concentrations in patients struggling with adherence, and therefore reduce the likelihood of breakthrough seizures after missed doses [13, 14]. However, PER’s efficacy in a nonadherence paradigm remains unknown. Ethical concerns over withholding medication from patients make this impossible to study in the clinic, meaning that the precise relationship between poor adherence and seizure incidence remains unknown and underscores the need for the present investigation.

Here we characterize the efficacy of PER when administered in a variably nonadherent medication-in-food paradigm to adult male rats with acquired spontaneous recurring seizures (e.g., epilepsy). We establish novel formulation methods to produce medication-in-food that is compatible with our novel automated delivery system to test the hypothesis that poor adherence results in worsened seizure control and to define the quantitative relationship between a missed dose and seizure incidence. These studies aim to further our understanding of the efficacy of mechanistically distinct ASM’s in a nonadherence paradigm and provide insight into whether certain ASM’s may be more forgiving than others following a missed dose or series of doses.

## Methods

### Animals

Upon arrival, adult male CD IGS Sprague Dawley rats (150–200 g; Charles River Laboratories, Wilmington, MA, USA) were allowed a 1-week acclimation period before the start of experimentation. All animals were group housed (5 rats/cage) in standard plastic cages under controlled environmental conditions in a vivarium on a 14:10 light/dark cycle and given ad libitum access to food and filtered water. Following kainic acid (KA) exposures (discussed below), animals were single housed in standard plexiglass cages with two types of enrichment (Nylabones and cardboard tubes) for the duration of the experiment.

### Kainic acid (KA) induced-status epilepticus (SE)

Status epilepticus (SE) was induced using a repeated low-dose KA administration paradigm as previously described [15]. All animals received an initial bolus of KA (10 mg/kg, i.p., Tocris, Bristol, UK) dissolved in sterile saline followed by a 1-h observation period. Animals then received subsequent doses of 5 mg/kg (i.p.) every 30 min until SE was reached—defined as having two generalized stage 4/5 Racine seizures within a 30 min period [16]. If an animal had a stage 5 seizure before the next 30 min dosing period, the subsequent dose was dropped to 2.5 mg/kg (i.p.) to prevent mortality. All animals were continuously monitored for seizure severity and duration for three hours following SE-onset. At the end of the observation period, all animals received a bolus of Lactated Ringers (3 mL, s.c. Baxter Int. Inc., Deerfield, IL, USA) to replace fluid loss and returned to their home cage. Pedialyte™-moistened chow and Napa Nectar hydration gel (Systems engineering Lab Group Inc., Napa, CA, USA) were provided as supportive care for 7 days post-SE or until animals returned to their pre-SE weight.

### Cortical EEG electrode implantation

Approximately 3–4 weeks following KA exposures, each rat was implanted with cortical EEG electrodes following surgical procedures outlined by the University of Washington IACUC rodent survival surgery guidelines. Twenty-four hours prior to surgery, animals received 2 mg Carprofen (p.o., Bio-Serv, New Jersey, USA) as prophylactic analgesia. On the day of surgery, animals were deeply anesthetized with isoflurane (1–3%, i.h.) in medical grade oxygen (flow rate = 1 L/min) and the head was stabilized using a stereotaxic apparatus. Ophthalmic ointment (Patterson Veterinary Supply Inc., St. Paul, MN, USA) was applied to the eyes and the surgical site was cleaned with alternating rounds of Betadine® scrub, and 70% isopropyl alcohol before administering a combined Lidocaine/Bupivacaine (1–2 mg/kg, s.c., Hospira, Lake Forest, IL, USA) injection for local anesthesia. A 1″ incision was made down the midline of the skull and a three-prong cortical electrode (MS333/1-A, P1 Technologies™, Roanoke, VA, USA) was implanted posterior to bregma with the two recording electrodes placed to the right of the sagittal suture and the ground electrode placed to the left. Four skull mount screws were placed on either side of the sagittal skull suture: two anterior and two posterior to bregma; the entire head mount was stabilized using dental acrylic. All rats were administered Carprofen (2 mg, p.o., Bio-Serv) for post-op analgesia and monitored daily during a 2-week recovery period.

### Video EEG (vEEG) recording and seizure detection

Approximately 8–10 weeks post KA-induced SE (Fig. 2), and 4–6 weeks post-surgery, animals were placed in individual custom-made plexiglass recording chambers (11″ × 16″ × 14″) and tethered to a rotating commutator (P1 Technologies™) for 24/7 vEEG monitoring. EEG data was acquired via a BioPac MP160/ EEC100 (BioPac Systems Inc., Goleta, CA, USA) system coupled to a pair of DVP 7020BE (Advantech, Milpitas, CA, USA) video capture cards. Custom software was used to synchronize video and EEG data while simultaneously controlling feeder system based on previously described methods [6]. Data was acquired in 12 h epochs, each recording epoch was analyzed using an unbiased two-step protocol: (1) seizure-like events were detected using an automated rodent seizure detection software, ASSYST (Kaoskey, Thornleigh Australia), where a seizure was defined as spike wave discharges with amplitude at least twice that of background activity in awake animals with at least 100 Hz frequency and duration of at least 10 s; (2) all events were manually confirmed by two trained investigators and seizures were scored based on a modified Racine scale [16] defined as (1) Mouth and facial clonus; (2) Head bobbing/nodding; (3) unilateral or bilateral forelimb clonus; (4) rearing; (5) rearing with falling, loss of righting reflex, or hindlimb extension. To adequately detect treatment-related effects on seizure control, each animal had to achieve an average > 1 stage 3 or higher seizure per week during their 4-week baseline recording period before enrolling into the 4-week intervention arm of the study. Any animal that did not reach enrollment criteria or lost their EEG implant over the course of the study was not included in the final analysis.

### PCBO and perampanel pellet formulation

Placebo (PCBO) food pellets were formulated in-house using a TDP5 desktop tablet press (LFA Machines, Dallas, TX, USA) equipped with a 12 mm modified ball punch and die tooling set. Formulated chow comprised of 96% (w/w) chocolate rodent chow (Bio-Serv, Flemington, NJ, USA), 2% (w/w) each of magnesium stearate (Sigma-Aldrich, St. Louis, MO, USA) and calcium silicate (Sigma-Aldrich), and < 0.1% (w/w) of filtered water. PER-containing pellets were formulated as described, except that 0.167 mg-PER/g-pellet was thoroughly mixed with the dry Bio-Serv chow before adding the remaining components. PER was generously provided by EISAI (Eisai Co Ltd, Tokyo, Japan) for this study.

### Quantitative analysis of PER in pellets and rat plasma

2-(1ʹ,6ʹ-dihydro-6ʹ-oxo-1ʹ-phenyl[2,3ʹ-bipyridin]-5ʹ-yl)-benzonitrile-d4 (perampanel-d4), MS-grade acetonitrile (ACN) and methanol (MeOH) were purchased from Fisher Scientific (Pittsburgh, PA). Formic Acid (FA) 88% ACS grade was purchased from Sigma Aldrich (St. Louis, MO). All stock drug solutions, buffers, and HPLC mobile phase were prepared using Milli-Q grade water (Millipore, Bedford, MA). All other consumables were purchased from Fisher Scientific (Pittsburgh, PA).

Chow pellets were prepared by cutting into 4 parts and pulverizing each quarter separately using a mortar and pestle. 15–25 mg of the pulverized pellet portion was weighed out and placed into individual Eppendorfs. 1 ml of ACN was added to each Eppendorf and vortexed for 10 min then centrifuged at 16 rcf for 5 min. 10 μl of the supernatant was then added directly into an autosampler vial containing 490 μl of ACN and 500 μl of H_2_O. 5 μl of the sample was injected onto the LC–MS platform. Chromatographic separation was achieved using a Water’s Acquity UPLC BEH C8, 2.1 × 50 mm, 1.7 µm column (Waters Corporation, Milford, MA, USA) using a gradient consisting of 0.1% formic acid in H_2_O (A) and 0.1% formic acid (FA) in acetonitrile (ACN) (B) at a flow rate 0.3 ml/min (See Table 1).

**Table 1:** LC Gradient for LC separation of perampanel

Time (min)	Flow (ml/min)	% A	% B
Initial	0.300	80	20
1.00	0.300	80	20
4.00	0.300	0	100
5.00	0.300	0	100
5.10	0.300	80	20

A: 0.1% formic acid in water; B: 0.1% formic acid in acetonitrile

**Fig. 1:**
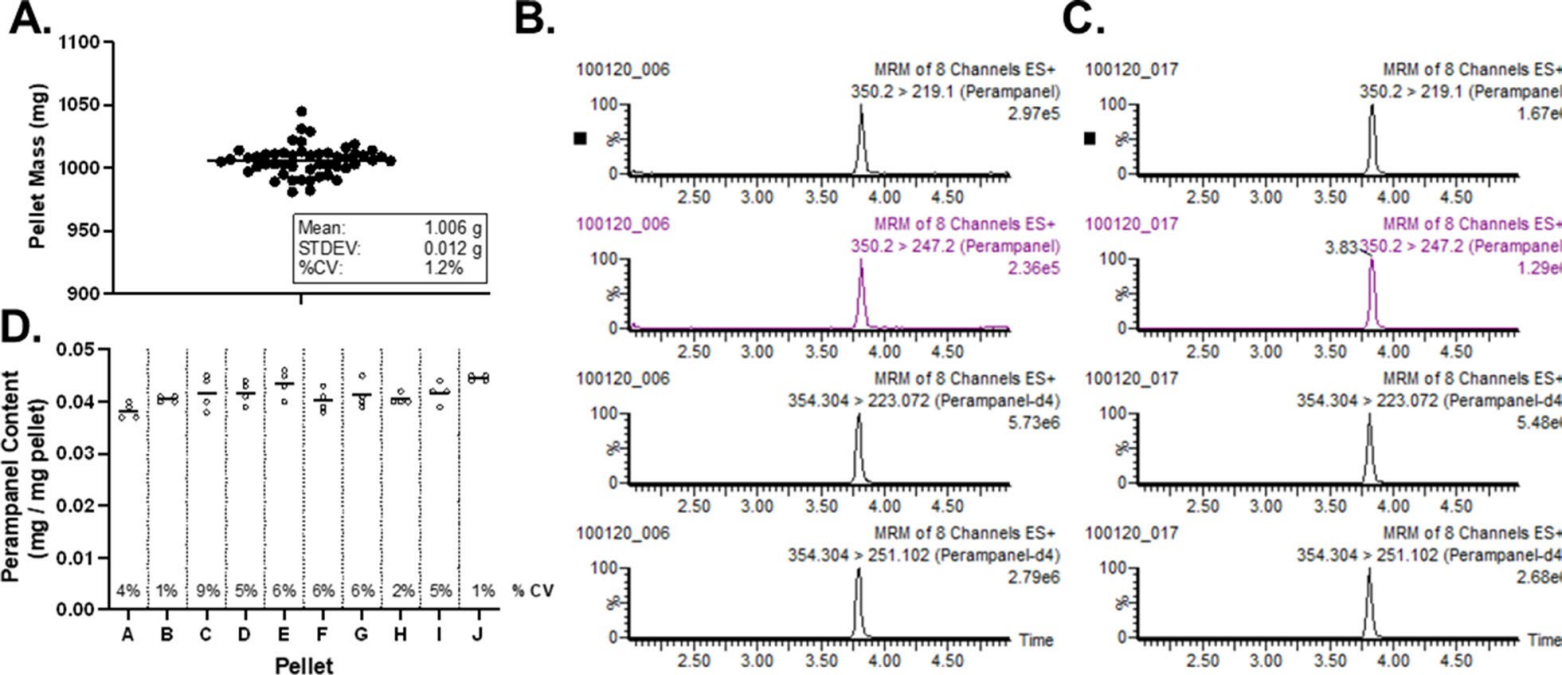
Novel formulation methods for drug-in-food generates consistent and uniform PER pellets. **A** Representative sample of 1 g PCBO pellets formulated in-house using a commercially available tablet press (TDP5, LFA Machines). Each data point represents a single pellet, n = 50 pellets. **B**, **C** Representative chromatogram for a 5 ng/ml calibrator sample (**B**) and subject sample (**C**). **D** PER content within and across a random sample of 10 individual food pellets (**A**–**J**). Each dot represents the PER content in ¼ of a pellet, with the line indicating mean value for each pellet. No significant differences between inter-pellet PER content as determined by one-way ANOVA

A Water’s Xevo-XS coupled to a Water’s I-Class Ultra high-pressure liquid chromatography system was used (Waters Corporation, Milford, MA, USA). Analytes were monitored in electrospray positive ionization mode (ESI+), using MRM mode. Instrument settings were as follows: Capillary (kV)—3.1, Source Temperature 150 °C, Desolvation Temperature 350 °C, Cone Gas Flow 150 l/Hr, Desolvation Gas Flow 1000 l/Hr, and Collision Gas Flow 0.16 ml/min. Two fragments for each analyte were used; one used for quantifying the other as a qualifier. A parent m/z of 350.2 and 354.2 fragmented to 247.2 and 251.1 was used for perampanel and parempanel-d4, respectively (see Table 2). An example of the lowest calibrator used as well as a subject sample can be found in Fig. 1B, C. Data was processed using MassLynxs (Milford, MA) and linear equations were formulated by using peak area ratios (PAR’s) allowing for 15% variability across calibrators and quality controls for acceptability.

**Table 2:** MS Transitions for perampanel quantitation

Compound	Parent (m/z)	Daughter (m/z)	Dwell time (s)	Cone (v)	collision (eV)
Perampanel	350.2	219.1	0.072	52	32
Perampanel	350.2	247.2	0.072	52	24
Perampanel-d4	354.3	223.1	0.072	52	36
Perampanel-d4	354.3	251.1	0.072	52	32

Internal Standard (I.S.) was purchased as a 100 μg/ml in MeOH of perampanel-d4 solution which was further diluted to 100 ng/ml in ACN. Calibration Curve and QC samples were prepared by making dilutions of stock solutions containing perampanel from 1 mg/ml stocks in MeOH and stored at − 20 °C. Calibration curves were created by analyzing drug-free plasma samples fortified with perampanel at 0, 5, 10, 50, 100, 500, 1000 ng/ml. Quality control (QC) samples in plasma were prepared at 50 and 500 ng/ml from separate dilutions of stocks than those used for the calibration curves. Neat solutions of perampanel were made in ACN for pellet concentration determination.

Plasma was deproteinated prior to LC–MS analysis. 10 μl of subject plasma, calibration, or quality control samples were pipetted into a 1.2 ml Eppendorf. 10 μl of internal standard consisting of permapanel-d4 (100 ng/ml in ACN) was added to each Eppendorf and vortexed for 5 s. 100 μl of ACN was then added to each Eppendorf and samples were vortexed for 15 s and then centrifuged at room temperature for 5 min at 16 rcf. 80 μl of supernatant was removed and placed directly into a 450 μl nunc 96 well plate and directly analyzed. 5 μl of the sample was injected onto the LC–MS platform. All LC–MS conditions for plasma evaluation were performed as described above.

### Oral dosing paradigm and plasma collection

Animals were maintained on a feeding regimen of 60 g/kg/day, delivered as 1 g pellets via a custom-built automated feeding system [7]. Upon the start of vEEG recording, animals were enrolled into a 4-week baseline period (weeks 1–4), receiving 60 g/kg/day PCBO chow at four equally spaced intervals throughout the day (15 g/kg, q.i.d). Upon reaching an average seizure frequency of 1 or more seizures/week (Racine Stage 3 or higher) for 4 weeks, animals were randomly enrolled to receive PER either 100% or randomly 50% of the time for 4 weeks. The 100% adherent group (n = 10) received an average daily dose of 10 mg/kg/day (2.5 mg/kg, q.i.d), while 50% adherent group (n = 12) received medicated pellets for only 50% of their meals at random over the course of 1-week intervals (~ 5 mg/kg/day). 10 mg/kg/day was determined to be the maximally tolerated oral dose when sub chronically delivered to age- and sex-matched naïve rats (7 days) (Additional file 1: Figure S1), the same dose with demonstrated efficacy in rodent acute seizure models [17]. The short half-life of PER in rodents (~ 1.7 h) ultimately informed the decision for q.i.d. dosing in order to maintain steady-state concentrations in fully adherent rats [17]. The number of medicated pellets was adjusted on a weekly basis to maintain a 10 mg/kg/day dosing regimen. Food consumption was monitored throughout the study and leftover pellets were discarded daily.

At 1-week intervals following initiation of PER treatment, blood samples were collected from the lateral tail vein approximately 1 h following a scheduled meal from all rats (100% and 50% groups). Blood samples were processed using microtainer tubes pre-coated with K2EDTA anticoagulant and separated using centrifugation (3000×*g* for 10 min). Plasma was transferred to individual microcentrifuge tubes and stored at − 80 °C until LC–MS analysis of PER content as described above.

### Study outcomes and statistical analyses

All EEG recording data was stored electronically and contained time-stamped events of feeding history (medicated vs. PCBO), seizure events, and detailed annotation of reviewer-identified seizure behavior. Data were analyzed and figures were generated using R version 4.0.3. (R Core Team, 2020) with the package ggplot2 [18], and GraphPad Prism (Version 9.0.0, La Jolla, CA, USA).

Primary outcomes for this study included seizure burden, a metric that summarizes the frequency and severity of seizures, as well as seizure frequency. Based on our previous data with CBZ in a nonadherence paradigm [5], sample size was powered to detect a twofold difference in average seizure burden between the two treatment groups with 80% power. Treatment-related effects on seizure burden or frequency was determined via 2-way ANOVA. Intergroup differences between study groups were compared using normalized values, e.g., each animal was normalized to its own baseline period and the groups compared to each other, via two-tailed Mann–Whitney test. Secondary outcomes evaluated changes in pellet consumption and systemic PER concentrations in plasma over time via mixed effects model with Geisser–Greenhouse correction. Results were reported as mean ± standard error of the mean (SEM) and significance was reported at level p < 0.05. The relationship between PER delivery, e.g., individual meal vs. patterns of acute adherence, and seizure occurrence via two-tailed binomial and Chi-square tests respectively. Results were reported as calculated odds ratio ± 95% confidence interval.

### Study approval

Experiments involving animals complied with the ARRIVE guidelines and were performed in accordance with the National Institutes of Health guide for the care and use of laboratory animals (NIH publication No. 8023, revised 1978) following protocols approved by the University of Washington, Institutional Animal Care and Use Committee (protocol 4387-01, approval date: 05/05/2019).

## Results

### Development of medication-in-food pellets and analytical quantification methods

Formulation of both medicated and PCBO pellets produced uniform, 1 g pellets that were consistent in size and shape (Fig. 1A). Daily administration of PCBO pellets to naïve Sprague Dawley male rats (60 g food/kg body weight/day) resulted in normal growth rates of animals as previously established [5, 6, 19]. Importantly, tandem liquid chromatography mass spectrometry bioanalytical methods were adapted from previous work [20, 21] for quantitation of PER content in both pellets and plasma. Chromatographic separation yielded high specificity and sensitivity with sharp, well-defined peaks in calibrator (Fig. 1B) and experimental plasma samples (Fig. 1C). Lower limit of quantification (LLOQ) was determined to be 5 ng/ml, which is significantly lower than expected plasma concentrations for our dosing range of 5–10 mg/kg/day p.o. [17]. Analytical quantification of PER content in formulated pellets resulted in consistent intra- and inter-pellet levels (Fig. 1D) that aligns with a total daily dose of 10 mg/kg/day, or 0.167 mg PER/g pellet when adjusted for 60 g/kg/day feeding regimen.

### Experimental schema and animal characteristics

The in-life portion of this study was completed over three independent cohorts with an initial enrollment of 50 animals. A total of 28 animals were lost to attrition from expected KA mortality (n = 22, 44% of total), loss of EEG head cap implant (n = 2, 4% of total), or failure to meet the enrollment criteria of ≥ 1 Racine Stage 3 or higher seizure/week to be included in the study (n = 4, 8% of total) [16]. By the end of the study, 22 remaining animals (cohort 1: n = 7; cohort 2: n = 7; cohort 3: n = 8) were randomly assigned to one of two experimental adherence groups: 100% PER (n = 10) or 50% PER (n = 12).

**Fig. 2:**
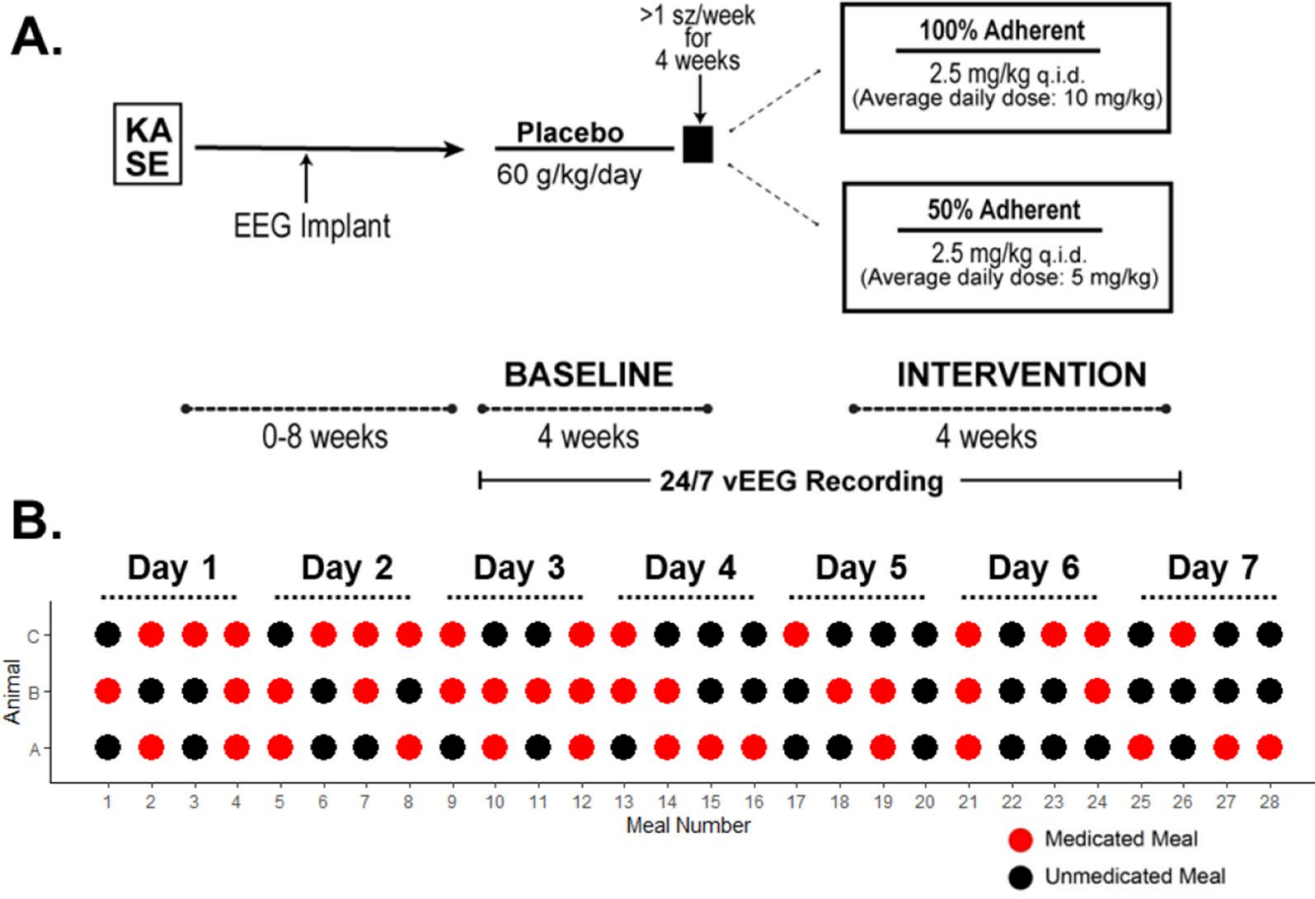
Experimental paradigm for measuring PER efficacy in a preclinical nonadherence paradigm. **A** Adult male Sprague Dawley rats (150–200 g) underwent repeated low dose kainic acid (KA)-induced status epilepticus (SE). Approximately 3–4 weeks following cessation of SE, rats were implanted with cortical EEG electrodes and allowed to fully recover before starting on PCBO chow at 60 g/kg/day (15 g/kg, q.i.d. p.o.). Baseline seizure frequency, burden and severity were recorded for 4 weeks; upon reaching an average > 1 Racine stage 3 or higher seizure/week animals randomly assigned to receive PER 100% of the time (average daily dose: 10 mg/kg/day) or randomly 50% of the time (average daily dose: 5 mg/kg/day) for a 4-week treatment phase. **B** Schematic depicting meal delivery over the course of a week (q.i.d. × 7 days) for 50% nonadherent treatment group. Red dots indicate medicated meal, black dot represents placebo meal

General characteristics of animals were the same across both groups, including baseline and study duration body weights, as well as general KA-SE characteristics (Additional file 2: Table S1). Animals in both groups met enrollment criteria (≥ 1 convulsive Stage 3 seizure/week) during a 4-week baseline recording period (starting at 8–10 weeks post-KA) and were enrolled onto PER at an average of 14.9 ± 1.9 weeks (100% PER) or 13.6 ± 1.1 weeks (50% PER) post-KA (Fig. 2A). Both groups received the same PER pellets (0.167 mg PER/g pellet) delivered on the same automated feeding schedule (15 g food/kg, q.i.d.) except that the nonadherent animals received medicated pellets at random only 50% of the time over the course of the week as depicted in Fig. 2B. PER was administered four-times daily since the half-life has been reported to be quite short in rodents (~ 1.67 h; [17]) and more frequent dosing is necessary to maintain steady state concentrations in fully adherent rats. No differences were found in weight gain (Additional file 2: Table S1) or pellet consumption (100% PER: 86 ± 4% or 50% PER: 85 ± 10%) between groups. Feeder errors were relatively uncommon, occurring only 12 times over the total 4,928 meals delivered in the study (< 0.2%).

### Overall degree of seizure control with PER is likely dose-dependent

**Fig. 3:**
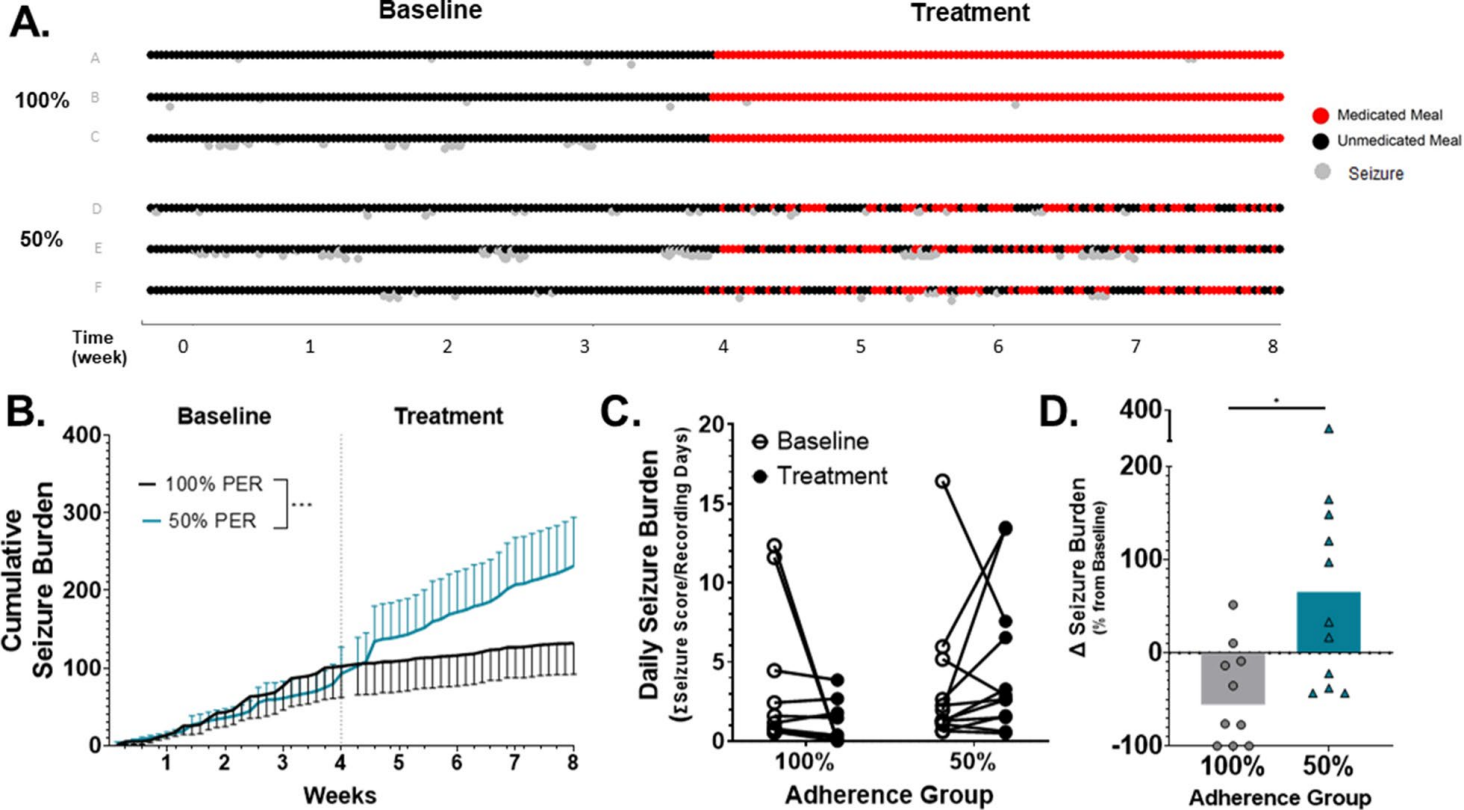
Full adherence to PER results in better seizure control than 50% variable nonadherence. **A** Representative plot demonstrating relationship between medication schedule (red & black dots) and seizure occurrence (gray dots) in three representative animals in the 100% (top) and 50% (bottom) adherence groups over baseline (week 1–4) and treatment periods (week 5–8). **B** Cumulative seizure burden in the 4-week baseline (PCBO) and 4-week treatment phases for 50% (teal) and 100% (gray) animals. Seizure burden = total sum of behavioral seizure scores. Data presented as mean ± SEM, PER: n = 10 (100%, gray), 12 (50%, blue); ***Significant time × treatment interaction (p < 0.001) as determined by repeated measures 2-way ANOVA with Geisser–Greenhouse correction. **C** Average daily seizure burden for individual rats during baseline (open circles) and intervention (closed circles) phase. Each dot represents single animal. Data not statistically significant at p < 0.05 as determined by 2-way ANOVA (top). **D** Normalized change in seizure burden compared to baseline in 100% (gray) and 50% (teal) adherent rats. Data presented as median ± 95% CI. *p < 0.05 as determined by two-tailed Mann–Whitney test

The impact of PER nonadherence on seizure control was evaluated relative to fully adherent animals over the duration of an 8-week study. Baseline seizure frequencies (Table 3) and seizure burden (Fig. 3B, C) were similar between experimental groups, albeit there was significant inter-animal variability within each group as represented in Fig. 3A. Addition of PER did not significantly change the average daily seizure frequency (Table 3) or average daily seizure burden (Fig. 3C) compared to baseline values in the either the 100% (mean ± SD: base: 3.63 ± 4.56 vs treat: 1.07 ± 1.34) or 50% (mean ± SD: base: 3.38 ± 4.24 vs. treat: 4.72 ± 4.59) groups. However, the cumulative seizure burden was significantly higher in nonadherent animals upon initiation of PER treatment [Fig. 3B; time x treatment interaction: F (55, 770) = 1.710, p < 0.001]. Given the significant inter-animal variability in seizure frequency in this model [15], seizure burden and frequency were normalized to each animal’s baseline period. When comparing between groups, fully adherent animals had a median reduction of 55% in their seizure burden compared to their untreated baseline phase [95% CI − 100%, 10%], which was significantly different from the 65% median increase [95%CI − 38%, 165%] in nonadherent animals (U = 21, p = 0.0089). Similar trends were observed in the changes in seizure frequency between treatment and baseline for both groups (Table 3). The 50% responder rate, or the number of animals that had greater than 50% reduction in seizure frequency, was 5/10 animals for fully adherent animals, while 0/10 animals in the variably nonadherent group reached this level of seizure control (Table 3). Similarly, 3/10 animals were completely seizure free in the 100% PER group, while 0/12 achieved seizure freedom in the 50% PER group.

**Table 3:** Summary of epilepsy seizure burden and responder rates for 100% and 50% PER groups

	100% adherent (~ 10 mg/kg/day) N = 10	50% adherent (~ 5 mg/kg/day) N = 12
Avg seizure frequency (# per day)		
Baseline	0.871 ± 1.115	0.747 ± 0.981
Treatment	0.246 ± 0.303	1.262 ± 1.352
Median Δ [95% CI]	− 50% [− 100, 51]	+ 54%* [− 43, 187]
> 25% responder rate #/n	5/10	5/12
> 50% responder rate #/n	5/10**	0/12
Seizure freedom #/n	3/10	0/12

Data presented as mean ± SD

^*^Significantly different at p < 0.05 as determined by Mann–Whitney test

^**^Significantly different at p < 0.01 as determined by Fishers exact test

**Fig. 4:**
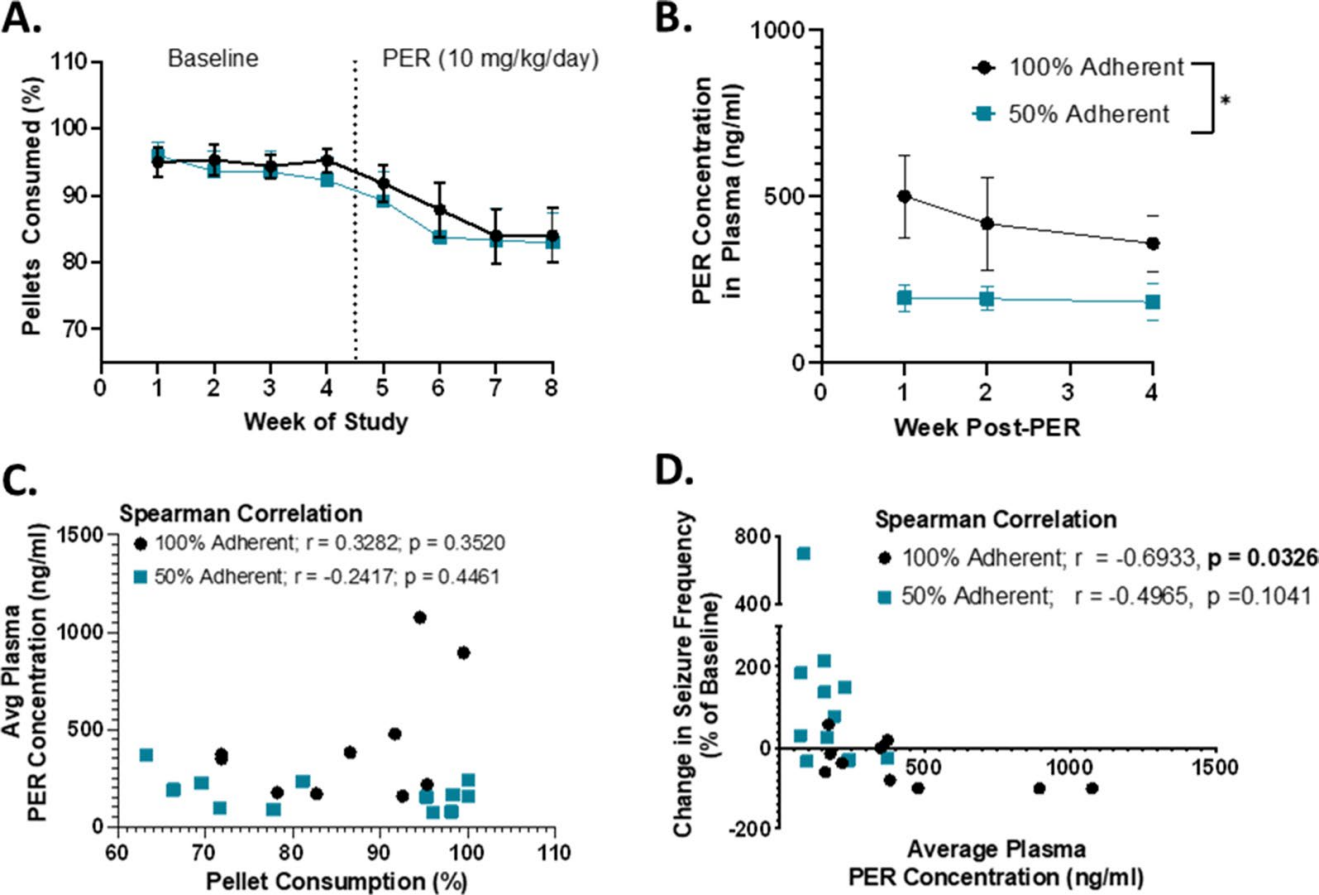
Overall degree of seizure control with PER is likely dose dependent. **A** Average weekly pellet consumption for animals in 100% group (black) and 50% adherent group (teal) during baseline (weeks 1–4) and treatment (weeks 5–8) periods. Data presented as mean ± SEM; n: 100% = 10; 50% = 12 animals). **Significant effect of time (p < 0.01) but no main effect of treatment (p = 0.7120) as determined by mixed effects model with Geisser–Greenhouse correction. **B** PER plasma concentrations at 1, 2, and 4 weeks following the start of PER chow in 100% (black) and 50% (teal) adherent groups. Data presented as mean ± SEM. *Significant effect if treatment at p < 0.05 as determined by mixed-effects model with Geisser–Greenhouse correction. **C** Spearman correlations for the relationship between the average pellet consumption and PER plasma concentrations for each animal over the 4-week treatment period in 100% (black) and 50% (teal) adherence groups. Each dot represents single animal. **D** Spearman correlation for the relationship between the change in seizure frequency (compared to baseline) and average PER plasma concentration for each animal in 100% and 50% adherence groups across the 4-week treatment period. *Significantly different at p < 0.05

The degree of seizure control between the two groups were unlikely due to differences in weekly pellet consumption as there was no significant main effect of treatment [Fig. 4A; F(1,20) = 0.1402, p = 0.7120] during baseline or treatment phases. Moreover, a significant effect of time [F(2.3, 46.6) = 12.66, p < 0.0001] but not time × treatment interaction [F(7,140) = 0.3790, p = 0.9134] suggests that changes in pellet consumption are likely due to factors that affect both groups equally, such as reduced metabolic rate because of age [22]. PER concentrations measured in plasma at 1-week sampling intervals following the start of treatment reveal that plasma concentrations were dependent on delivered daily dose since PER concentrations in the 100% group were about twice as high as the 50% group [Fig. 4B; F (1,20) = 6.126, p = 0.02]. Importantly, there was no significant time effect [(F (1.9, 23.9) = 0.2834, p = 0.7457], suggesting that rats in both groups had consistent plasma levels over the duration of the study, which were not significantly correlated with weekly pellet consumption (Fig. 4C). Moreover, the degree of seizure control, or change in seizure frequency compared to baseline, was negatively correlated with PER plasma levels in fully adherent animals, highlighting that seizure control was associated with greater PER levels in plasma [Fig. 4D, r = − 0.6933, p = 0.03]. While nonadherent animals trended toward the same relationship, this did not reach statistical significance (r = − 0.4965, p = 0.1).

### Acute patterns of nonadherence are not associated with increased risk of breakthrough seizures

To quantify the relationship between a missed dose and subsequent seizure, we analyzed the distribution of meals occurring before every seizure in nonadherent animals (Table 4). Of note, one or more seizures occurring between mealtimes, e.g., seizure cluster, was collapsed into a single event for analysis. Being that the addition of PER did not change seizure cluster phenotype or frequency in either fully adherent or nonadherent animals, nor was there a significant relationship between any meal pattern and the risk of a seizure cluster, clusters were collapsed into single events as to not unequally weight certain meal patterns over others (Additional file 3: Figure S2). The medication delivery schedule was analyzed for a total of 206 seizure events across 12 animals as previously described [6]. For instance, the distribution of PCBO vs. PER delivery was tabulated for every meal preceding a seizure for each animal (e.g., one meal before, two meals before, three meals before etc.) and the distribution was represented as the percentage of the total of delivered meals (Table 4). For example, if there was no relationship between a single missed dose and the incidence of a seizure, the expected distribution would be 50:50 (PCBO vs. PER) for every meal preceding a seizure. A two-tailed binomial test did not identify any individual meal that deviated from the expected distribution, although the third meal before a seizure trended towards being unmedicated (i.e., 44% of meals were delivered as PER for the third meal, p = 0.0828). Together, these data suggest there is no relationship between a single missed dose of PER and incidence of a seizure.

**Table 4:** Expected vs. observed distribution of PER meals (%) before a seizure

Meal before a seizure	% Medicated			Statistical Significance
	Expected	Observed	95% CI	p value
1	50	52	45–58	0.6259
2	50	50	43–57	> 0.999
3	50	44	37–51	0.0828
4	50	54	47–61	0.2960
5	50	54	47–61	0.2960
6	50	47	41–54	0.4435
7	50	46	39–52	0.1854
8	50	51	44–57	0.8895
9	50	52	45–58	0.7277
10	50	52	45–58	0.7277

Data presented as % of meals that were medicated with PER before a seizure. The expected distribution is 50:50. A total of 206 seizure events were analyzed. No statistically significant differences as determined by two-tailed binomial test

**Fig. 5:**
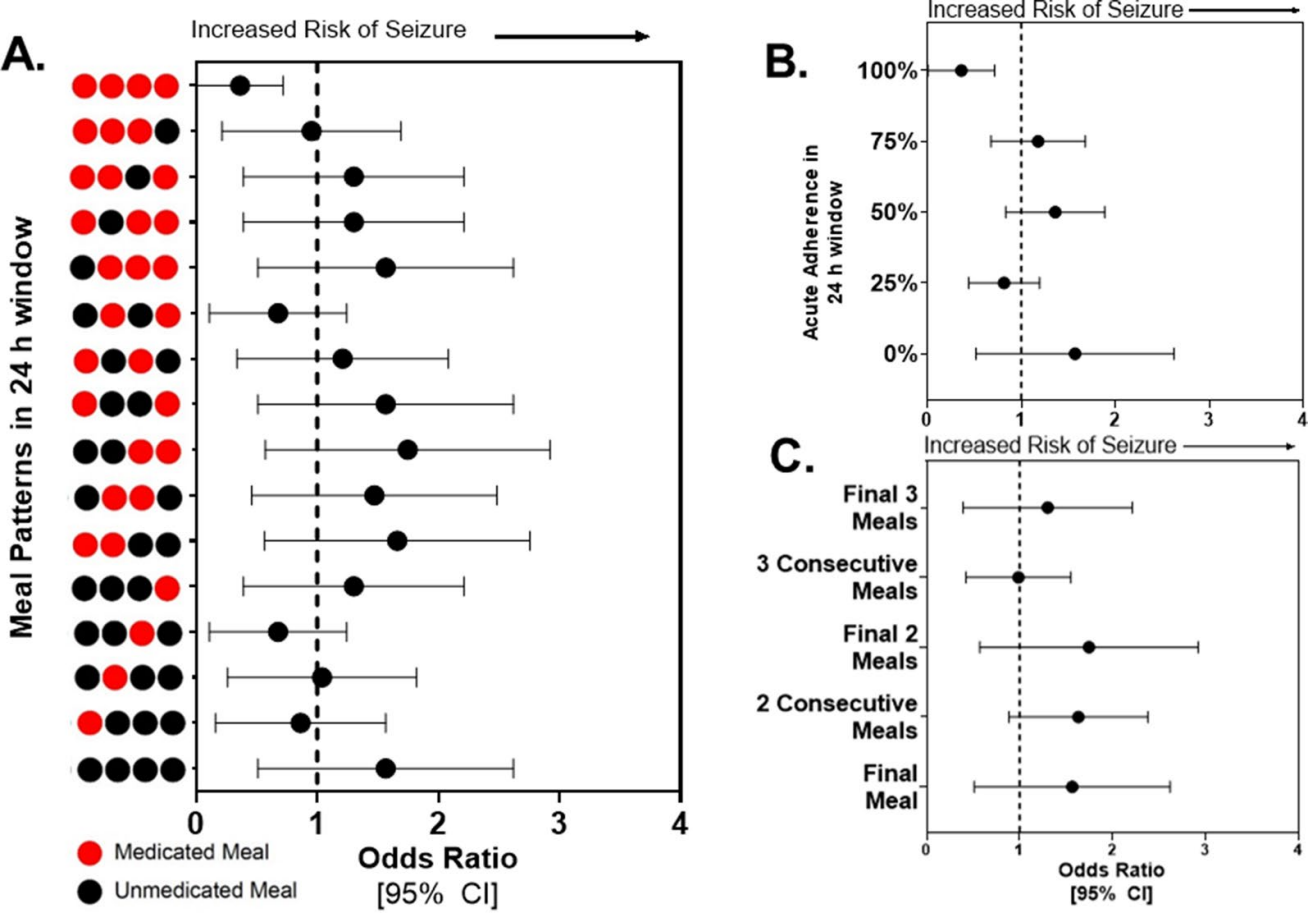
24-h periods of nonadherence is not associated with increased risk of breakthrough seizures. **A** Calculated odds ratio for the relationship between individual patterns of PER nonadherence in a 24 h window (y-axis) and incidence of a seizure (x-axis). Black: PCBO; Red: PER. Data presented as the odds ratio ± 95% CI. Any CI that does not cross one indicates a significant relationship between a specific meal pattern and seizure incidence as determined by two-tailed Chi-Square analysis. n = 206 seizure events across 12 animals. **B** Odds ratios computed for the relationship between patterns of acute adherence (y-axis) and the association with a seizure. Data presented as the odds ratio ± 95% CI. Any CI that does not cross one indicates a significant relationship between a pattern of acute nonadherence and seizure incidence as determined by Chi-Square analysis. **C** Odds ratios computed for the relationship between scenarios of acute nonadherence (y-axis) and the occurrence of a seizure. Data presented as the odds ratio ± 95% CI. Any CI that does not cross one indicates a significant relationship between a pattern of acute nonadherence and seizure incidence as determined by Chi-Square analysis

From a clinical perspective, medication nonadherence can take various forms across different individuals. For instance, some may forget a single dose at sporadic times, while others may have short, sustained periods of nonadherence. Therefore, in addition to quantifying the relationship between a single missed dose of PER and seizure occurrence, we also evaluated whether acute periods of nonadherence within a 24-h window were associated with seizure incidence. Based on q.i.d. dosing, there are 16 possible patterns of medication delivery that range from completely nonadherent to fully adherent (Fig. 5A). We did not identify any meal patterns associated with increased incidence of a seizure, including 24 h periods where PER was missed for every dose (OR [95% CI] 1.335 [0.6448, 2.716], z = 0.754, p = 0.4482). However, we found that the odds of seizure occurrence were lower following periods of full adherence within 24 h (OR [95% CI] 0.2194 [0.0658, 0.7658], z = 2.55, p = 0.011). Moreover, when grouping meal patterns into acute periods of adherence (e.g., 0, 25, 50, 100%), we similarly found no association between acute nonadherence and seizure incidence (Fig. 5B). Likewise, when evaluating different scenarios of nonadherence within a 24 h window, e.g., 2 consecutive doses are missed, or the final 3 doses before a seizure were missed (Fig. 5C), there was no situation that resulted in an increased risk of seizure incidence. Taken together, these data highlight an unexpected relationship between medication delivery and breakthrough seizures suggesting that a missed dose, or series of missed doses of PER within a 24-h period may not significantly increase the risk of unexpected seizures.

## Discussion

Herein, we describe a study where we have successfully formulated drug-in-chow pellets that are uniform in size, shape, and ASM content, which are compatible with our automated feeder delivery system utilized for long-term drug studies in preclinical research [5–7]. While useful across all disease types, we utilized our novel formulation methods to expand on previous work with CBZ [5, 6] to determine how nonadherence to a mechanistically distinct first-line therapy for focal seizures, perampanel (PER), impacts seizure control in an etiologically relevant animal model of acquired epilepsy.

Medication-in-food delivery approaches are not novel for chronic drug studies in preclinical models of epilepsy [19, 23, 24]. However, few studies outside of our own have taken advantage of an automated medication-in-food delivery system to deliver ASM therapy on a schedule that supports pharmacokinetic-based dosing [5, 6]. Limitations of such systems require pellets to be formulated in a way that is compatible in both size and shape with the system and investigators may be limited to the pre-determined selections that are offered from commercial vendors. Moreover, commercial vendors may not have the regulatory license in place to manufacture food pellets with controlled substances such as PER, which is listed as a Schedule III agent in the U.S. Drug Enforcement Agency (DEA) Controlled Substances Act [25]. We overcame these hurdles by using commercially available tooling resources that can be customized to create pellets of any shape and size. Moreover, our formulation methods generate pellets with uniform medication content that can be replicated with any compound of interest. Together, these methods may reduce the barriers to chronic drug studies and provide an essential tool for improving preclinical drug discovery across all models of chronic diseases.

These results support the hypothesis that poor ASM adherence results in worsened seizure control. One interpretation may be that seizure control is dose-dependent and that the 50% group may not have reached a large enough dose of PER to render any antiseizure effects. It is worth noting that PER levels in both the 100% and 50% animals remained stable and were well within the range of effective concentrations in adult patients that respond to PER therapy (425 ± 270 ng/ml, [26]). Moreover, our data also highlight that any 24 h period where PER was administered for all four doses was associated with a lower risk of breakthrough seizures, suggesting that our findings in nonadherent animals cannot fully be explained by dose alone. These data do not rule out the possibility that increasing the dose of PER beyond 10 mg/kg/day may have resulted in better seizure control in the refractory animals. However, the goal of ASM therapy is to attain seizure freedom without untoward side effects [27], and early pilot data in age- and sex-matched naïve rats suggest that motor impairment and sedation occur at oral doses as low as 20 mg/kg/day. One might imagine the clinical implications of pushing the dose into an intolerable range; e.g. increased adverse events could perpetuate nonadherent behavior, rendering any dose-dependent improvement in efficacy negligible. It is not surprising that few animals attained seizure freedom given that the post-KA model of chronic epilepsy more closely resembles human TLE [28], which is the most refractory type of epilepsy (~ 30% of patients achieve seizure freedom with adequate therapy [29]). Other groups have determined that the natural history of epilepsy is progressive in untreated animal models, likely due to the continued brain damage from uncontrolled seizures [15, 30]. Therefore, it is not surprising that we witnessed a progressive worsening of seizure frequency increased in nonadherent rats that were unable to attain seizure control. While this may seem to differ from what one might expect in the patient, there are a few key differences that should be considered. Rather than initiating treatment soon after the onset of a reported seizure, which is standard clinical practice [31], we started PER therapy in animals with established epilepsy that may have been inherently more refractory. Moreover, unlike in clinical practice when the dose is often titrated to find adequate seizure control [31], we used a fixed-dose dosing paradigm, which certainly highlights an important limitation to be addressed in future investigations. Nonetheless, many features of the post-KA rat emphasize strengths of this model to support further investigation into whether medication nonadherence may contribute to future pharmacoresistance with different ASM therapies, a limitation that was not addressed by the present investigation.

One of the most surprising findings from this study is the apparent lack of pharmacokinetic dependence between a missed dose of PER and breakthrough seizures. Given the short half-life of PER in rodents (~ 1.67 h), one would expect that a missed dose (i.e., 6 h, ~ 4 half-lives) would result in PER concentrations that fall below therapeutic levels, leading to breakthrough seizures [17], which was not the case in our animals. Interestingly, PER concentrations remained consistent in the 50% group at levels that have been reported to be effective in patients. One limitation is that our therapeutic drug monitoring was completed at 1-week intervals, and while PER concentrations did not fluctuate over time, we do not have brain or plasma measurements within any acute 24-h period to confirm how PER concentrations change with different patterns of adherence and whether this correlates with breakthrough seizures. This is an ongoing line of investigation. Interestingly, PER concentrations modestly declined over time in fully adherent rats, albeit these changes were not statistically significant. While some ASM’s can induce their metabolism (e.g., CBZ), to our knowledge, this has not been reported for PER in patients or animal studies [32, 33] and is not something we would expect to be underlying this effect but instead may be due to age-related decline in pellet consumption over the duration of the study [22]. Nonetheless, these findings raise an interesting possibility that missing a dose of PER may not negatively impact seizure threshold despite changes in drug concentrations within the local microenvironment.

On the surface, variable adherence to PER differs from published work with CBZ [5, 6]. While a missed dose of PER does not increase the risk of breakthrough seizures, a missed dose of CBZ may lead to unexpected seizures much later in time (e.g., days later), even if subsequent doses have been taken. Granted, the CBZ studies initiated nonadherence at the time of “epilepsy diagnosis” in animals, which is relevant for newly diagnosed patients who may be more likely to adopt nonadherent behavior [34], but makes it difficult to interpret the impact of nonadherence on disease progression. For instance, it is unclear whether CBZ nonadherence resulted in an improvement or worsening of seizure clusters, and whether those seizure clusters were accounted for in the analyses of the relationship between missed dose and seizure events. Conversely, we found that PER nonadherence did not alter disease phenotype, allowing us to limit our analyses to the relationship between missed dose(s) and seizure events (e.g., 1+ seizure occurring between meal periods). Despite the differences in study design and analysis, both CBZ and PER highlight an interesting non-pharmacokinetic relationship between ASM nonadherence and breakthrough seizures that should be considered in clinical practice.

The non-pharmacokinetic relationship between PER nonadherence and breakthrough seizures raises interesting questions about the mechanisms underlying this phenomenon. It is entirely possible that PER may have lasting pharmacodynamic effects on neuronal networks mediated through AMPAR, which may translate to prolonged increases in seizure threshold. Previous reports suggest that AMPAR antagonists may have a neuroprotective role by preventing neuronal death and inhibiting epileptogenesis [35, 36]. Other studies have reported lasting effects of PER treatment on spike wave discharges and behavioral comorbidities in animals, even for months after the drug had been cleared from circulation [35]. While certainly beyond the scope of the present study, it is possible that PER may have protective but temporary effects on network hyperexcitability to prevent the immediate risk of breakthrough seizures, despite declines in drug concentrations.

If substantiated in patients, the clinical implication of our work supports consideration of a patient’s adherence profile when undergoing ASM selection, rather than only selecting an ASM that meets the appropriate tolerability and efficacy profile for a given patient’s seizure type [37]. A long-half life and favorable pharmacokinetic profile reduce barriers for attaining optimal adherence [13], however, certain ASM’s that are less likely to result in breakthrough seizures because of missed dose(s) may be an important consideration for patients at higher risk of nonadherence. If our preclinical data is substantiated in the clinic, PER’s favorable pharmacokinetic profile in humans (t_1/2_ ~ 105 h) [33], combined with a lowered risk of breakthrough seizures suggests that it may provide a certain forgiveness factor if a dose is missed within a 24-h window. It is worth noting that, to date, preclinical studies have only investigated the relationship between nonadherence and seizure control using male subjects. While clinical data suggest that men may be more prone to adopting nonadherent behavior [3], further investigation is warranted into whether sex as a biological variable changes the relationship between adherence and breakthrough seizures.

Patterns of medication nonadherence may not always be random and may take different forms that are influenced by affordability, accessibility, or other patient-specific factors (i.e., perceived lack of benefit, intolerable adverse events etc.) [3, 34, 38]. While this study reflects the impact of random nonadherence, we recognize the value of approximating other human treatment paradigms more closely. For example, brief drug holidays, or prolonged periods where patients abstain from ASM therapy represent other patterns of nonadherence; however, there is very little clinical or preclinical data to suggest whether these periods may increase the risk of conferring treatment resistance later on when therapy is restored [39, 40]. Preclinical work with CBZ suggests that a brief drug holiday does not necessarily predispose an animal to developing CBZ resistance when adherence is restored [6], but whether this generalizes to ASM’s across different mechanistic classes is unknown and warrants further investigation. We also recognize the need for future studies to better capture the nuanced and dynamic behavior of medication adherence as it relates to therapy changes and other clinical decision making. For instance, what might happen in a patient who is initially adherent but may over time become less diligent in their behavior and end up with more frequent breakthrough seizures. Clinical practice may dictate a switch in monotherapy or escalation to polytherapy after a loss of seizure control, however, the consequences of such changes without first addressing adherence remain unknown. Nonetheless, these kinds of questions are the foundation as to why preclinical models, such as the one presented herein, are so critical for advancing our understanding of complex relationships between medication nonadherence and breakthrough seizures.

## Conclusions

The unique experimental approach presented herein highlights the important relationship between medication adherence and seizure control, which may provide clinically meaningful insights into ASM management in patients with epilepsy. These results suggest that a missed dose of the mechanistically novel ASM, PER, does not immediately increase the risk of breakthrough seizures in rats with acquired epilepsy. If upheld in the clinic, our findings highlight a non-pharmacokinetic relationship between PER nonadherence and breakthrough seizures that have important implications for clinical practice.

### Supplementary Information

**Additional file 1: Figure S1.** Dose-Finding Study in Naïve Male Age-Matched Sprague Dawley Rats. **A.** Schematic of 10-day dose finding study. Animals were habituated to automated feeder system with 3-days of placebo feeding (15 g/kg, q.i.d.) before randomizing to 1 of 4 treatment groups: PCBO, 5, 10, 20 mg/kg/day of PER. Minimal motor impairment (MMI) was evaluated at baseline, and 3- and 7-days after the initiation of PER treatment. MMI defined as the sum score of 5 criteria, each evaluated on a scale of 1 (completely impaired) to 4 (no impairment). Criteria included: home-cage activity, gait, grip reflex, hind limb extension, righting reflex. **B.** Daily pellet consumption over 10-day study. Significantly different from all treatment groups at **p < 0.01 as determined by repeated measures 2-way ANOVA with Tukey’s multiple comparisons.** C**. Total MMI score for all rats at baseline, 3- and 7-days post PER treatment. Data presented as mean ± SEM. Significantly different at *p < 0.05, **p < 0.01 as determined by 2-way ANOVA with Dunnet’s Multiple Comparisons; n = 4/group.

**Additional file 2: Table S1.** Demographic and Baseline Characteristics of Animals Enrolled in Study.

**Additional file 3: Figure S2.** PER does not alter seizure cluster phenotype when administered in a fully adherent or variably adherent paradigm. **A.** Percentage of animals that present with seizure clusters (e.g., 2 + seizures occurring between mealtimes or every 6-h) during baseline or treatment periods in the 100% (gray, n = 7) and 50% (teal, n = 12) animals. **B.** Total percentage of seizure burden that forms as cluster (2 + seizures within 6-h) during baseline and treatment periods for 100% (gray) and 50% (teal) groups. Data presented as mean ± SEM. Ns = not significantly different as determined by repeated measures 2-way ANOVA. **C.** Total # of cluster episodes during baseline and treatment periods for 100% (gray) and 50% (teal) groups. Data presented as mean ± SEM. Ns = not significantly different as determined by repeated measures 2-way ANOVA **D.** Odds ratios computed for the relationship between patterns of acute adherence and the occurrence of a seizure. Data presented as the odds ratio ± 95% CI. Any CI that does not cross one indicates a significant relationship between a missed meal and a seizure as determined by Chi-Square analysis. Only those animals with seizures during baseline and treatment periods included in analysis.

## Data Availability

The data presented in this study are available on request from the corresponding author and presented as individual datapoints in all accompanying tables and figures in this manuscript.

## Abbreviations

ASM: Antiseizure medicine
CBZ: Carbamazepine
CNS: Central nervous system
KA: Kainic acid
PCBO: Placebo
PER: Perampanel
SD: Standard deviation
SE: *Status epilepticus*
SEM: Standard error of the mean
TLE: Temporal lobe epilepsy
vEEG: Video electroencephalography
