# Supplementary material for: Perampanel’s forgiveness factor in a variable medication adherence paradigm in a rat model of chronic epilepsy
Source: J Transl Med. 2023 Sep 20;21:642. doi: 10.1186/s12967-023-04490-z (PMC10510183; doi:10.1186/s12967-023-04490-z)
Supplement: Supplementary file 1 — Additional file 1: Figure S1. Dose-Finding Study in Naïve Male Age-Matched Sprague Dawley Rats. A. Schematic of 10-day dose finding study. Animals were habituated to automated feeder system with 3-days of placebo feeding (15 g/kg, q.i.d.) before randomizing to 1 of 4 treatment groups: PCBO, 5, 10, 20 mg/kg/day of PER. Minimal motor impairment (MMI) was evaluated at baseline, and 3- and 7-days after the initiation of PER treatment. MMI defined as the sum score of 5 criteria, each evaluated on a scale of 1 (completely impaired) to 4 (no impairment). Criteria included: home-cage activity, gait, grip reflex, hind limb extension, righting reflex. B. Daily pellet consumption over 10-day study. Significantly different from all treatment groups at **p < 0.01 as determined by repeated measures 2-way ANOVA with Tukey’s multiple comparisons. C. Total MMI score for all rats at baseline, 3- and 7-days post PER treatment. Data presented as mean ± SEM. Significantly different at *p < 0.05, **p < 0.01 as determined by 2-way ANOVA with Dunnet’s Multiple Comparisons; n = 4/group. [file 12967_2023_4490_MOESM1_ESM.pdf]

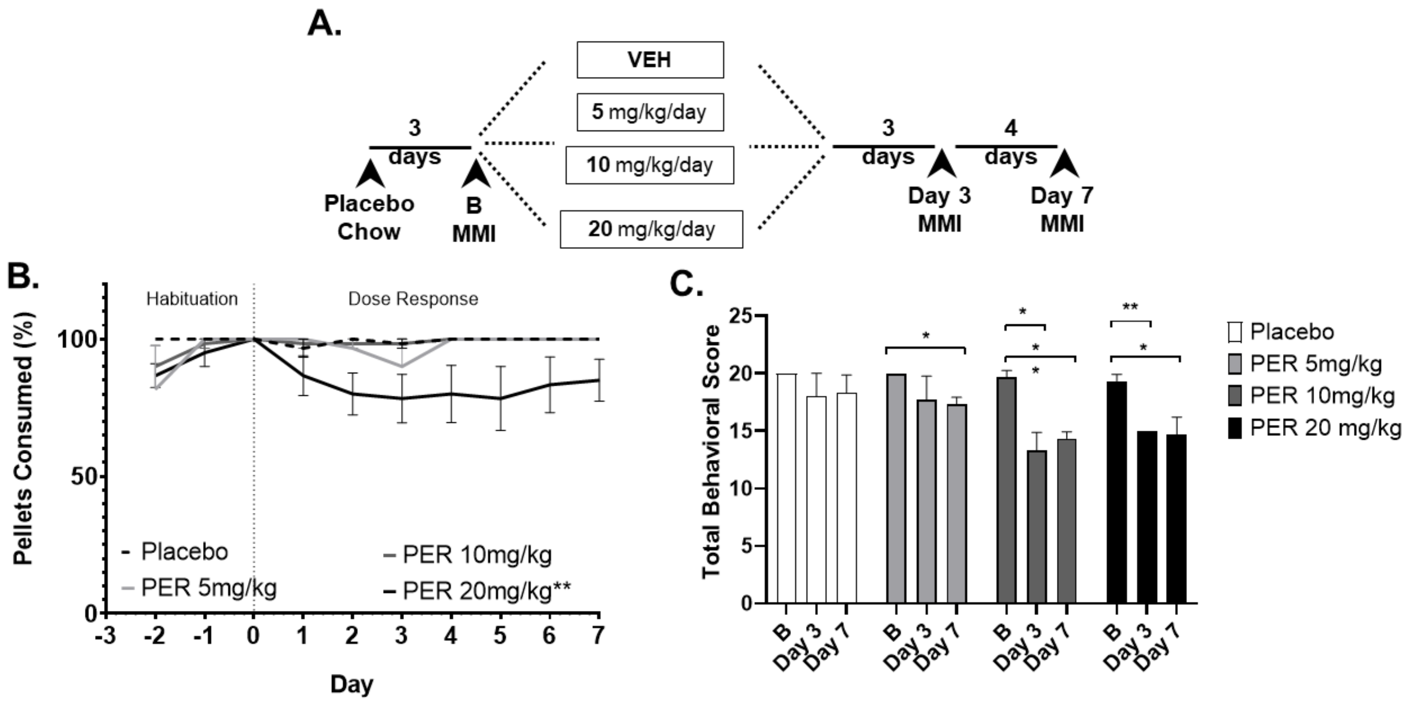


**Additional File 1: Figure S1: Dose-Finding Study in Naïve Male Age-Matched Sprague Dawley Rats. A.** Schematic of 10-day dose finding study. Animals were habituated to automated feeder system with 3-days of placebo feeding (15 g/kg, q.i.d.) before randomizing to 1 of 4 treatment groups: PCBO, 5, 10, 20 mg/kg/day of PER. Minimal motor impairment (MMI) was evaluated at baseline, and 3- and 7-days after the initiation of PER treatment. MMI defined as the sum score of 5 criteria, each evaluated on a scale of 1 (completely impaired) to 4 (no impairment). Criteria included: home-cage activity, gait, grip reflex, hind limb extension, righting reflex. **B.** Daily pellet consumption over 10-day study. Significantly different from all treatment groups at $^{**}p<0.01$ as determined by repeated measures 2-way ANOVA with Tukey's multiple comparisons. **C**. Total MMI score for all rats at baseline, 3- and 7-days post PER treatment. Data presented as mean ± SEM. Significantly different at $^{*}p<0.05$, $^{**}p<0.01$ as determined by 2-way ANOVA with Dunnet's Multiple Comparisons; n=4/group
