# Supplementary material for: Perampanel’s forgiveness factor in a variable medication adherence paradigm in a rat model of chronic epilepsy
Source: J Transl Med. 2023 Sep 20;21:642. doi: 10.1186/s12967-023-04490-z (PMC10510183; doi:10.1186/s12967-023-04490-z)
Supplement: Supplementary file 2 — Additional file 2: Table S1. Demographic and Baseline Characteristics of Animals Enrolled in Study. [file 12967_2023_4490_MOESM2_ESM.pdf]

Additional File 2: Table S1: Demographic and Baseline Characteristics of Animals Enrolled in Study

	**100% Adherent** (~10 mg/kg/day) N = 10	**50% Adherent** (~5 mg/kg/day) N =12	**Attrition** (mortality, head cap loss) N = 28
**Weight** (g)			
Kainic Acid SE	209 ± 9.5	211 ± 11	214 ± 11
Baseline (end)	647 ± 68.75	651 ± 64.85	--
Treatment (end)	710 ± 94	701 ± 72	--
**Kainic Acid Dose** (mg/kg)	19 ± 7.7	20 ± 7.8	20 ± 5.5
SE Score[¥]	2.827 ± 0.324	2.938 ± 0.228	3.787 ± 0.881*
**Enrollment onto PER** (weeks post-KA)	14.9 ± 1.9	13.6 ± 1.1	--

[¥]Seizure Score defined as average Racine Score

*Significantly different from 100% and 50% groups at $p<0.05$ as determined by Kruskal-Wallis test with Dunn's multiple comparisons

Data presented as mean ± SD.

PER: perampanel; N = number of animals; KA = kainic acid
