# Supplementary material for: Perampanel’s forgiveness factor in a variable medication adherence paradigm in a rat model of chronic epilepsy
Source: J Transl Med. 2023 Sep 20;21:642. doi: 10.1186/s12967-023-04490-z (PMC10510183; doi:10.1186/s12967-023-04490-z)
Supplement: Supplementary file 3 — Additional file 3: Figure S2. PER does not alter seizure cluster phenotype when administered in a fully adherent or variably adherent paradigm. A. Percentage of animals that present with seizure clusters (e.g., 2 + seizures occurring between mealtimes or every 6-h) during baseline or treatment periods in the 100% (gray, n = 7) and 50% (teal, n = 12) animals. B. Total percentage of seizure burden that forms as cluster (2 + seizures within 6-h) during baseline and treatment periods for 100% (gray) and 50% (teal) groups. Data presented as mean ± SEM. Ns = not significantly different as determined by repeated measures 2-way ANOVA. C. Total # of cluster episodes during baseline and treatment periods for 100% (gray) and 50% (teal) groups. Data presented as mean ± SEM. Ns = not significantly different as determined by repeated measures 2-way ANOVA D. Odds ratios computed for the relationship between patterns of acute adherence and the occurrence of a seizure. Data presented as the odds ratio ± 95% CI. Any CI that does not cross one indicates a significant relationship between a missed meal and a seizure as determined by Chi-Square analysis. Only those animals with seizures during baseline and treatment periods included in analysis. [file 12967_2023_4490_MOESM3_ESM.pdf]

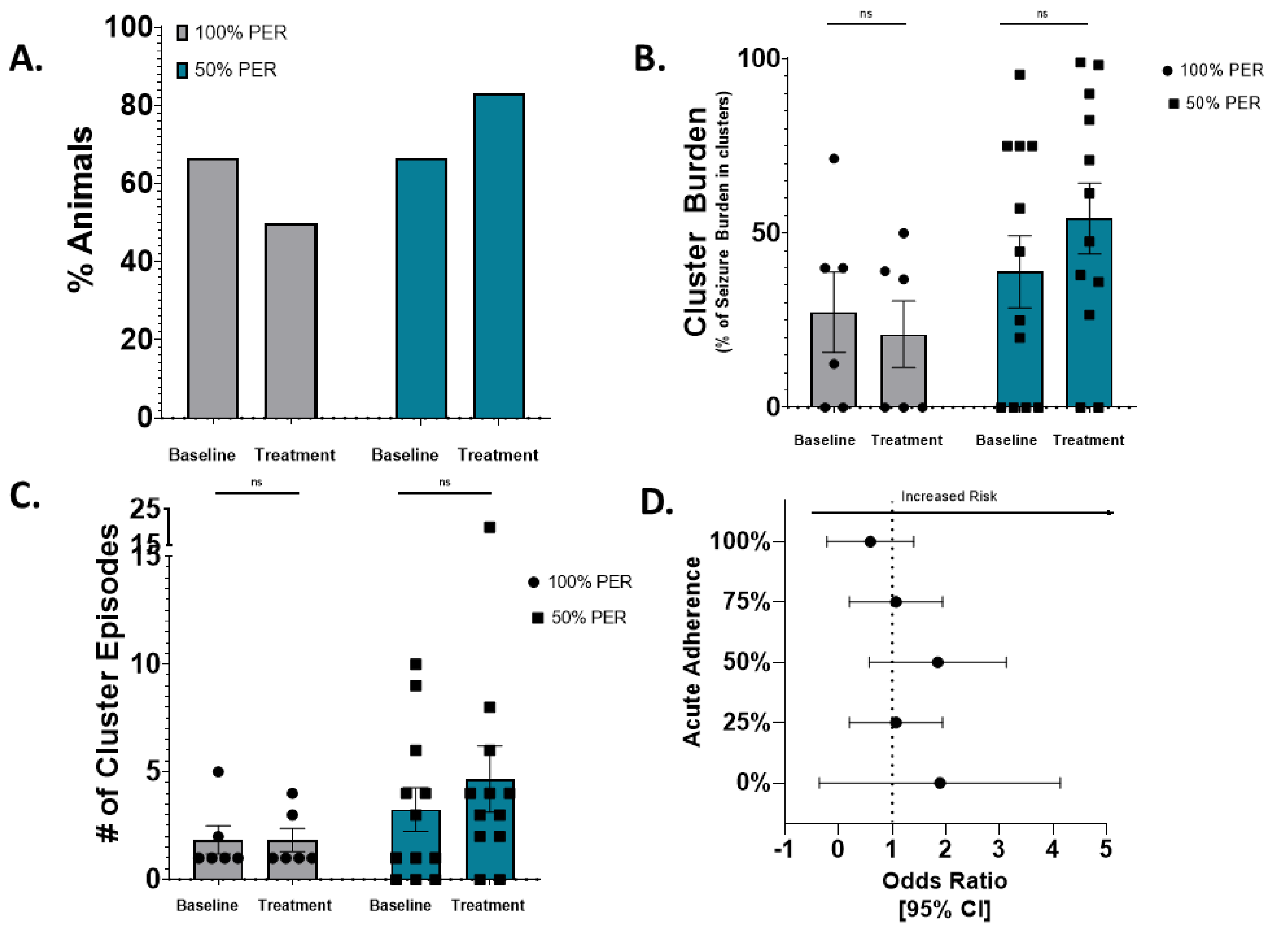

Additional File 3: Figure S2: PER does not alter seizure cluster phenotype when administered in a fully adherent or variably adherent paradigm. **A.** Percentage of animals that present with seizure clusters (e.g., 2+ seizures occurring between mealtimes or every 6-hours) during baseline or treatment periods in the 100% (gray, n=7) and 50% (teal, n=12) animals. **B.** Total percentage of seizure burden that forms as cluster (2+ seizures within 6-hour) during baseline and treatment periods for 100% (gray) and 50% (teal) groups. Data presented as mean ± SEM. Ns = not significantly different as determined by repeated measures 2-way ANOVA. **C.** Total # of cluster episodes during baseline and treatment periods for 100% (gray) and 50% (teal) groups. Data presented as mean ± SEM. Ns = not significantly different as determined by repeated measures 2-way ANOVA **D.** Odds ratios computed for the relationship between patterns of acute adherence and the occurrence of a seizure. Data presented as the odds ratio +/- 95% CI. Any CI that does not cross one indicates a significant relationship between a missed meal and a seizure as determined by Chi-Square analysis. Only those animals with seizures during baseline and treatment periods included in analysis.
